# Vancomycin Susceptibility within Methicillin-resistant Staphylococcus aureus *L*ineages

**DOI:** 10.3201/eid1005.030556

**Published:** 2004-05

**Authors:** Robin A. Howe, Alastair Monk, Mandy Wootton, Timothy R. Walsh, Mark C. Enright

**Affiliations:** * Southmead Hospital, Bristol, United Kingdom; † University of Bath, Bath, United Kingdom; ‡ University of Bristol, Bristol, United Kingdom

**Keywords:** vancomycin, methicillin resistance, *Staphylococcus aureus* epidemiology

## Abstract

Methicillin-resistant *Staphylococcus aureus* (MRSA) with reduced vancomycin susceptibility (VISA, vancomycin-intermediate *S. aureus*) has been reported from many countries. Whether resistance is evolving regularly in different genetic backgrounds or in a single clone with a genetic predisposition, as early results suggest, is unclear. We have studied 101 MRSA with reduced vancomycin susceptibility from nine countries by multilocus sequence typing (MLST) and characterization of SCC*mec* (staphylococcal chromosomal cassette *mec*) and *agr* (accessory gene regulator). We found nine genotypes by MLST, with isolates within all five major hospital MRSA lineages. Most isolates (88/101) belonged to two of the earliest MRSA clones that have global prevalence. Our results show that reduced susceptibility to vancomycin has emerged in many successful epidemic lineages with no clear clonal disposition. Increasing antimicrobial resistance in genetically distinct pandemic clones may lead to MRSA infections that will become increasingly difficult to treat.

Methicillin-resistant *Staphylococcus aureus* (MRSA) is a major problem around the world, causing hospital-acquired infections and, more recently, infections in the community (*1*,*2*). The glycopeptides, particularly vancomycin, have been the mainstays of therapy for MRSA, and the emergence of resistance to these agents is of great concern.

The first *S. aureus* with reduced vancomycin susceptibility (vancomycin MIC ≥8 μg/mL) was isolated in 1997 (*3*,*4*), and similar isolates have since been discovered in several countries. These vancomycin-intermediate *S. aureus* (VISA) isolates are relatively rare; a recent review found 21 VISA described in the literature (*5*). However, strains of *S. aureus* have been described that are vancomycin-susceptible by conventional testing but have a subpopulation of resistant cells. These heterogeneous VISA (hVISA) are more common; reports from around the world indicate that 0.5%–20% of MRSA are heteroresistant (*5*). The clinical importance of hVISA is debatable, but evidence shows that they are precursors of VISA, and they have been implicated in treatment failure in deep-seated infections (*6*,*7*).

A study of early VISA strains that used multilocus sequence typing (MLST) and analysis of the SCC*mec* region suggested that they were all descended from the New York/Japanese (8*,**9*) pandemic MRSA clone (*10*); the first high-level vancomycin-resistant isolates that have acquired the *vanA* gene cassette from enterococci are also members of this clone (F. Tenover, pers. comm.). Researchers have suggested that isolates of the New York/Japanese pandemic MRSA clone may be predisposed to become vancomycin resistant, perhaps because of loss-of-function mutations in the *agr* (accessory gene regulator) gene (*11*). We analyzed the genetic backgrounds of a geographically diverse sample of VISA and hVISA to investigate the evolutionary history of such strains.

## Materials and Methods

We collected 101 isolates of MRSA with reported heterogeneous or homogeneous resistance to vancomycin (MIC ≥ 8 mg/L) from China (n = 1), France (31), Japan (2), Norway (14), Poland (13), Sweden (1), United Kingdom (28), and the United States (11). Antimicrobial susceptibility tests were performed by the agar dilution method of the National Committee for Clinical Laboratory Standards. Isolates were described as VISA if they fulfilled the three criteria adopted by the Centers for Disease Control and Prevention, that is, broth microdilution vancomycin MIC of 8 to 16 mg/L, MIC ≥ 6 mg/L on E-test, and growth on brain-heart infusion agar containing 6 mg/L vancomycin (*12*). Isolates with heterogeneous resistance to vancomycin were confirmed by using population analysis profiling followed by measuring the area under the curve (PAP-AUC), as described previously (*13*). The prototypic hVISA strain MU3 was used as a standard, and isolates with an AUC ≥0.9 compared to MU3 were described as hVISA.

MLST was performed as described previously (*10*). The seven housekeeping gene sequences were compared to known alleles in the MLST database (available from: http://www.mlst.net), and the resulting allelic profiles (which define sequence types, STs) were used to interrogate the databases for matches within records of the 988 isolates held there. The MLST databases contain molecular and epidemiologic data on *S. aureus* isolates from carriage and disease, including examples of all major MRSA clones (*10*). Data from this study were added to the *S. aureus* MLST database, and the entire dataset was analyzed by using the BURST algorithm to assign isolates to clonal complexes (CCs), which are lineages containing genetically related isolates (sharing 100% genetic identity at ≥5/7 loci used). Polymerase chain reaction (PCR) analysis of the *ccr* (chromosomal cassette recombinase) and *mec* (methicillin resistance) regions was performed to discriminate the four main SCC*mec* types (I–IV) on the basis of combinations of the two regions. Conventional PCR was used to detect SCC*mec* I–III by using the primers described in Ito et al. (*14*) and SCC*mec* IV by using those described by Daum et al. (*15*). These results were confirmed using the multiplex method of Oliveira et al. (*16*). Detection of *agr* subgroups I–IV was performed by PCR of the region surrounding *agrD*, which codes for an autoinducing peptide, according to the method of Peacock et al. (*17*).

## Results and Discussion

The results are shown in the Table. PAP-AUC values for the isolates varied from 0.9 to 3.01 and 91/101 isolates were designated hVISA on the basis of a PAP-AUC value ≥0.9. Nine isolates were designated as VISA.

**Table:** Characteristics of study isolates with reduced vancomycin susceptibility^a^

Genotype			Clonal type	*agr* type	Vancomycin resistance phenotype (no. of strains)	PAP-AUC	Country of origin	Antimicrobial susceptibility^*†^				
CC	ST	SCC*mec*						Lzd	Syn	Gen	Cip	Rif
5	5	I	EMRSA-3	II	hVISA (1)	0.98	UK	S	S	R	R	R
				I	VISA (1)	1.9	USA	S	S	R	R	S
5	5	II	New York/Japanese	II	hVISA (10)	0.97–1.23	Japan, Sweden, France, Poland, UK, USA, Norway	S	S	S/R	S/R	S/R
				I or II	VISA (3)	1.4–1.92	USA	S	S	S	R	S/R
5	5	IV	Pediatric	I or II	hVISA (3)	1.19–1.32	UK	S	S	S	S/R	S/R
5	5	NT		I	VISA (1)	1.44	France	S	S	R	R	R
8	8	I		II	hVISA (3)	0.92–1.32	France, UK, Norway	S	S	R	R	R
8	8	II	Irish-1	II	hVISA (3)	1.04–1.2	France, USA, Norway	S	S	S/R	R	S/R
8	8	IV	EMRSA-4, -6	I	hVISA(11)	0.94–1.24	France, USA	S	S/R	S/R	R	S/R
22	22	IV	EMRSA-15	I or II	hVISA (7)	0.9–1.25	UK	S	S	S	R	S/R
25	25	NT		I	hVISA(1)	1.13	UK	S	S	R	R	R
30	36	II	EMRSA-16	II	hVISA (3)	0.92–1.17	UK	S	S	R	R	R
45	45	II		I	hVISA (1)	1	USA	S	S	R	R	S
8	239	I or II	Brazilian/Portuguese	I or II	hVISA (10)	0.9–1.22	France, Poland, China, Norway, UK	S	S	R	S/R	S/R
				I	VISA (3)	1.44–3.01	France, Poland, UK	S	S/R	R	R	S/R
8	239	NT		I	hVISA (1)	0.92	France	S	S	R	R	R
8	246	NT		I	hVISA (1)	1.13	Norway	S	S	R	R	R
8	247	I	Iberian	I	VISA (1)	1.57	UK	S	S	R	R	R
				I or II	hVISA (37)	0.9–1.33	France, Poland, UK, Norway	S	S	R	R	S/R

^a^S, susceptible; R, resistant; NT, nontypeable; PAP-AUC, population analysis profiling followed by measuring the area under the curve; Lzd, linezolid; Syn, synercid; Gen, gentamicin; Cip, ciprofloxacin; Rif, rifampin; CC, clonal complex; ST, sequence type; EMRSA, methicillin-resistant *Staphylococcus aureas* found in the United Kingdom (UK) ; hVISA, heterogeneous vancomycin-intermediate *S. aureus*; USA, United States of America.

From the genotyping results, strains were divided into clonal complexes, which can be subdivided according to sequence type (ST) and SCC*mec* differences. The clonal complexes CC5, CC8, CC22, CC30, and CC45 represent the five pandemic MRSA lineages that have been previously described (*10*). Our results show that hVISA has arisen in all five of these pandemic clones and that VISA has so far developed in CC5 and CC8. The three most common MRSA clones present in the United Kingdom (EMRSA-3, EMRSA-15, EMRSA-16) (*18*) are included within these lineages, and reduced vancomycin susceptibility has been identified in all of these clones. All lineages displayed resistance to multiple antimicrobial classes, and only the new oxazolidinone linezolid was active against all strains.

Only *agr* subgroups (alleles) I and II were found in isolates in this study with 7/9 VISA and 57/92 hVISA having *agr* I. Within the 14 clones in this study, the proportion of isolates with particular *agr* alleles was variable. The presence of both *agr* I and *agr* II among VISA/hVISA, even in genetically similar isolates, suggests that the genes for the *agr* system are horizontally transferred. Sakoulas et al. reported an association of *agr* II with the development of vancomycin resistance (*11*). Our results show that VISA/hVISA also emerged in strains with *agr* I.

Molecular analyses of VISA isolates to date have focused on isolates from the United States and Japan, and results have indicated that all strains belong to the New York/Japanese MRSA clone. In our study, we found that hVISA isolates have emerged from every lineage that has produced pandemic MRSA clones, and VISA isolates have emerged in two of five lineages, in all likelihood from hVISA precursor isolates.

Increasing drug resistance in clones that are multidrug resistant and adapted to spread and cause serious disease can do much damage in the modern hospital environment. We have shown that reduced vancomycin susceptibility has emerged in genetically and phenotypically diverse MRSA clones throughout the world. This finding suggests that vancomycin resistance has the potential to become a widespread problem in MRSA strains already resistant to multiple antimicrobial agents.
